# Enhanced Hypercoagulability in Sickle Cell Anaemia Patients with Chronic Leg Ulcers

**DOI:** 10.1155/2020/5157031

**Published:** 2020-11-22

**Authors:** David Sackey, Yvonne Dei-Adomakoh, Edeghonghon Olayemi

**Affiliations:** ^1^Haematology Unit, Komfo Anokye Teaching Hospital, Kumasi, Ghana; ^2^Department of Haematology, University of Ghana Medical School, Accra, Ghana; ^3^Ghana Institute of Clinical Genetics, Korle Bu, Accra, Ghana

## Abstract

Sickle Cell Anaemia (SCA) is associated with a hypercoagulable state resulting in a predisposition to venous thromboembolism. With improvements in the quality of care, more patients with SCA survive into adulthood with an associated increase in the frequency of end-organ damage and chronic complications such as chronic leg ulcers (CLUs). These ulcers rarely occur in the first decade of life and are recurrent, painful, and slow-to-heal. This study tested the hypothesis that coagulation is enhanced in SCA patients with CLU. 145 participants (50 SCA with CLU, 50 SCA without CLU, and 45 with haemoglobin AA) were assessed to determine their coagulation profile using selected tests of coagulation. The SCA with the CLU group had the lowest mean haemoglobin (Hb) concentration. SCA patients with and without CLUs had elevated mean platelet counts, shorter mean aPTT, and marginally prolonged mean PT  compared to HbAA patients. SCA with CLUs patients had a significantly shortened aPTT than those without CLUs (*p* = 0.035) and HbAA (*p* = 0.009). There were significant differences in the mean PT between SCA with CLUs patients and HbAA (*p* = 0.017); SCA without CLU and HbAA (*p* = 0.014). SCA with and without CLUs patients had higher mean D-dimer levels compared to HbAA. There was a negative correlation between Hb concentration and duration of CLU (*r* = -0.331, *p* = 0.021). In conclusion, our study demonstrates a heightened hypercoagulability in SCA patients with CLUs. We did not test for platelet activation, and it is not clear what role, if any, the enhanced hypercoagulability plays in the pathogenesis of CLUs in SCA. It will be useful to ascertain if antiplatelet agents or/and anticoagulants quicken the healing of CLUs in SCA patients.

## 1. Introduction

Sickle Cell Anaemia (SCA) is associated with an increase in prothrombotic factors and a decrease in physiologic anticoagulants, resulting in a predisposition to venous thromboembolism [1]. With improvements in the quality of care, more patients with SCA survive into adulthood with an associated increase in the frequency of end-organ damage and chronic complications such as leg ulcers [2]. SCA patients may develop recurrent, painful, slow-to-heal chronic leg ulcers (CLU). The prevalence of CLU in SCA varies geographically, affecting 75% of SCA patients in Jamaica but only 8–10% of North American patients [3] and 10–19% in Ghana [4, 5]. CLU in SCA rarely occurs before 10 years of age [5, 6].

These ulcers run an indolent course; they heal up to 16 times slower than venous ulcers [7], and about 97% of healed sickle CLUs will recur in less than 1 year [3]. Due to the chronic nature of these ulcers, SCA patients with CLUs may experience significant disfigurement, social isolation, and loss of income [3]. The increased disability, absence from work or school, and high utilization of healthcare resources severely affect the quality of life of individuals with SCA and CLUs [8, 9]. Earlier, Ankra-Badu (1992) reported the mean duration of a sickle cell CLU in Ghana as three and half years [10], a more recent study obtained a mean duration of 4.6 years [5]. This is similar to what is obtained in the United States [11].

The pathogenesis of CLU in SCA is complex and may include mechanical obstruction of blood vessels by dense sickled red cells, venous incompetence, bacterial infections, abnormal autonomic control with excessive vasoconstriction, in situ thrombosis, lupus anticoagulant, haemolysis, anaemia, hypoxia, and decreased nitric oxide bioavailability leading to impaired endothelial function have all been proposed as potential contributory factors [10–12]. Thrombosis may lead to chronic damage to the blood vessel. This impedes blood flow in the microcirculation causing CLUs. [3]. A study conducted in Nigeria showed that SCA patients with CLUs are more likely to have lupus anticoagulant than those without CLUs. They also had a significantly longer kaolin clotting time than those without CLUs [13]. In this current study, we tested the hypothesis that coagulation is enhanced in SCA patients with CLUs.

## 2. Materials and Methods

This was a cross-sectional study. Participants were recruited from the sickle cell disease clinic located at the Ghana Institute of Clinical Genetics, Korle Bu, Ghana, a referral health facility that receives adolescent and adult patients with sickle cell disease from across Ghana, with an average daily attendance of about fifty patients [14].

### 2.1. Study Participants

Participants were recruited from among known sickle cell anaemia (SCA) patients (confirmed by Hb electrophoresis), aged 12 years and above with CLUs. SCA patients without leg ulcers in steady-state (from the same clinic) and healthy individuals with Hb AA phenotype recruited from among voluntary blood donors at the Accra Area Blood Transfusion Centre of the National Blood Service, Ghana, served as controls. For the purpose of this study, steady-state was defined as the absence of acute painful crises, intercurrent illness, or any change due to therapy (including blood transfusion) in the previous 4 weeks before recruitment [15]. Written informed consent was obtained from participants or their guardians/parents (if the participant was a minor). Participants on anticoagulant drugs, antiplatelet agents such as aspirin, oral contraceptives, or who were pregnant were excluded.

### 2.2. Data and Blood Sample Collection

Using a data extraction form, the following were extracted from the participants' medical records: age, sex, age when the first CLU occurred, duration of leg ulcer, and number of times the ulcer reoccurred (if any).

A 7.5 mL venous blood sample was collected with minimal stasis from the antecubital fossa of each participant. Three millilitres of blood was put in an EDTA specimen bottle. This was used for automated Full Blood Count (FBC) using the Horiba ABX Micros ES60 analyzer following the manufacturers' instruction, and Hb electrophoresis using the Cellulose Acetate Method as previously described [16]. The remaining blood sample (4.5 mL) was added to 0.5 mL of trisodium citrate in another specimen bottle and was used for coagulation tests. Prothrombin time (PT), activated partial thromboplastin time (aPTT), and fibrinogen concentration (Clauss method) were measured as previously described [17]. D-dimer was assayed using Helena Auto Blue D-dimer 400 automated immunoturbidimetric assay, following the manufacturers' instructions.

### 2.3. Data Analysis

Data were summarized as means ± standard deviation for continuous variables and percentages for categorical variables. The mean difference across various categories of variables was analyzed using ANOVA, with Bonferroni post hoc analysis for pairwise comparison. The association between clinical parameters of study participants and coagulation profile parameters were analyzed using Spearman's correlation. The analysis was done with Statistical Package for Social Sciences (SPSS) software, version 20 (IBM Incorporated). A *p*-value less than 0.05 was considered statistically significant. Approval for this study was obtained from the College of Health Sciences, the University of Ghana, Ethical and Protocol Review Committee: MS-Et/M.7-P4.3/2012-13.

## 3. Results

A total of 145 participants were enrolled: 50 SCA with CLU, 50 SCA without CLU, and 45 with HbAA phenotype. The SCA with CLU group had the lowest mean Hb concentration. Post hoc analysis showed a significant difference in mean Hb concentration between the SCA with CLU and SCA without CLU groups (*p* = 0.038), and that SCA without CLU participants were younger than SCA with CLU and HbAA participants (*p* = 0.003), Table 1.

The earliest age at which ulceration of the leg occurred in this study population was 8 years, and the latest was 49 years.

### 3.1. Coagulation Profile of Study Subjects

Table 2 shows the coagulation profile of the study groups. SCA patients with and without CLU had elevated mean platelet counts, shorter mean aPTT, marginally prolonged mean PT compared to HbAA participants. Post hoc analyses among the groups showed significant differences in the mean values between the study groups: SCA with CLU had a significantly shortened aPTT than those without CLU (*p* = 0.035) and HbAA (*p* = 0.009). However, there was no significant difference observed between the SCA without CLU and HbAA groups (*p* = 0.605). With respect to platelet count, there was no significant difference in the mean counts among both SCA groups (*p* = 0.206). However, they had significantly higher mean counts compared to the HbAA group (*p* < 0.001).

Post hoc (Bonferroni) analyses showed significant differences in the mean PT between SCA with CLU and HbAA group (*p* = 0.017) and the SCA without CLU and HbAA control group (*p* = 0.014). There was no significant difference in the PT between SCA patients with and without CLU, *p* = 0.960.

In Table 3, SCA with and without CLU groups had higher mean D-dimer levels compared to the HbAA group. Post hoc (Bonferroni) analyses for D-dimer levels showed no significant difference between the levels seen in both SCA groups (*p* = 0.559) and between the SCA patients without CLU and HbAA (*p* = 0.588). However, there was a significant difference in the D-dimer levels between SCA with CLU and HbAA group (*p* = 0.032).

The association between selected characteristics of the study participants and coagulation markers are shown in Table 4. There was a negative correlation between the frequency of recurrence of CLUs and platelet counts (*r* = - 0.311, *p* = 0.028) and between Hb concentration and duration of the CLU (*r* = -0.331, *p* = 0.021). There was a positive correlation between age and duration of CLU (*r* = 0.351, *p* = 0.015). There was a negative correlation between Hb level and platelet counts (*r* = -0.664, *p* < 0.001), D-dimer levels (*r* = −0.233, *p* = 0.005), and PT (*r* = -0.233, *p* = 0.005). Also, the Hb of the participants seems to increase as the aPTT approaches normal (*r* = 0.185, *p* = 0.026).

## 4. Discussion

Our study agrees with previous reports that CLUs in SCA patients occur at a relatively older age, and that few cases occur before the age of 10 years [5, 6]. Also, SCA patients with CLU in our study had significantly lower Hb concentration compared to those without CLU. This supports the earlier finding associating CLU in SCA with haemolysis and severe anaemia [8].

### 4.1. D-Dimer Concentration

Elevated D-dimer levels have been reported in SCA (HbSS) patients compared to HbAA controls [17]. Similarly, in this study, D-dimer levels were higher in SCA with CLU compared to HbAA controls. However, there was no significant difference in the mean D-dimer l evels between the SCA without CLU group and the group with CLU 1.04 mg/mL vs. 1.56 mg/mL, *p* = 0.559. This may be due to the fact that D-dimers have been shown to be elevated in SCA patients in steady-state and during painful crises [18]. Though mean D-dimer levels in our study were comparable to those reported by Fakunle et al. [19] in steady-state (1.32 mg/mL); we were unable to demonstrate a similar significant difference between SCA patients without CLU in steady-state and the HbAA group (*p* = 0.588). However, our study showed a significant difference in D-dimer concentration of SCA with CLU and HbAA (*p* = 0.032). Sickle Cell Anaemia (SCA) patients with and without CLUs have more active coagulation and fibrinolytic systems. This may be a response to increased haemolysis in SCD, especially in those with CLUs [8, 20]. An increased level of D-dimer is an indication of increased fibrin formation and an active fibrinolytic system. Our study also showed an inverse relationship between D-dimer levels and Hb concentration. This again may be a result of the increase in haemolysis associated with CLU [21].

### 4.2. Fibrinogen Levels in the Study Groups

Mean fibrinogen concentration in our SCA groups was higher: 314.3 ± 109.83 and 284.90 ± 83.46 mg/dL for SCA with and without CLU, respectively, than values reported by Buseri et al. [22]. Among steady-state HbSS patients in Nigeria (220 ± 30 mg/dL), this may be due to different assay technique used we used the Clauss method in our study while Buseri et al. used the clot weight method.

Nsiri et al., working with a relatively small sample size (*n* = 12), in contrast to our study and Buseri et al. [22], reported significantly lower fibrinogen concentration in SCA patients in the steady-state than Hb AA controls 96.5 ± 17.0 mg/dL vs. 102.5 ± 11.5 mg/dL, *p* < 0.001 [23].

### 4.3. aPTT and PT in Subjects

Sickle cell anaemia patients with CLU had a significantly shortened aPTT compared to those without CLU (*p* = 0.014) and HbAA participants (*p* = 0.002). However, aPTT for the SCA without CLU group was similar to that of the HbAA controls (*p* = 0.605). This is in keeping with the assertion that CLUs in SCA are characterized by a hypercoagulable state [24]. The finding from this study, however, is in contrast to findings from other studies. In a study by Buseri et al., in Nigeria [25], a significantly prolonged aPTT was recorded (46.0 ± 9.6 seconds, *p* < 0.001) among steady-state HbSS patients. In contrast to Buseri et al. [22], aPTT values provided by Nsiri et al. [23] in Tunisia was 34.4 ± 4.2 seconds and was quite similar to the values obtained in this study among SCA subjects without CLU. An isolated elevation of factor VIII concentration has previously been reported in SCD patients, and it may explain the shortened aPTT [23, 26–28]. The shortened aPTT indicates a highly active coagulation system and thus a hypercoagulable state which could lead to thrombosis; thrombosis could lead to venous congestion and subsequently venous hypertension which can facilitate the development and sustenance of a leg ulcer.

There was a statistically significant difference in mean PT among our study groups (*p* = 0.006), with the HbAA group having a shorter PT (Table 2). This may occur due to a reduction in the concentration of factor *V* which has earlier been reported in SCD patients [23, 26]. This reduction will produce a normal or slightly prolonged PT instead of the expected shortening. Our findings agree with earlier studies from Sudan, where a normal PT was reported by Omer et al. [29].

### 4.4. Platelets Count and Leg Ulcers

Platelet counts were also significantly elevated in both SCA groups (*p* < 0.001) compared to controls. Platelets are activated in SCD during the steady-state and further activation is seen during acute pain episodes [30]. It has also been reported that circulating platelets in SCD patients are chronically activated, and platelet aggregation is increased [31, 32]. This may be attributed to the increased numbers of young, metabolically active platelets or increased plasma levels of platelet agonists, such as epinephrine, adenosine diphosphate or thrombin, in the blood of SCD patients [33]. These factors might contribute to hypercoagulability in SCA patients [34]. As earlier suggested, hypercoagulability may be responsible for CLUs, either indirectly as a consequence of venous thrombosis or directly by thrombus formation in small arteries, arterioles, capillaries, or venules [34].

## 5. Conclusion

This study demonstrates a heightened hypercoagulability in SCA patients with CLU above the baseline hypercoagulability, which is known to be present in patients with SCD even in steady-state. Thus, though selected markers of coagulation were significantly different between SCA with and without CLU and HbAA groups, they were often significantly higher in the SCA with CLU group. Though we did not test for platelet activation in our study participants and it is not clear what role, if any, the enhanced hypercoagulability plays in the pathogenesis of CLUs in SCA. The prevalence of leg ulcer in SCA patients varies geographically; it is possible that this is due to genetic factors which may affect the concentration of various clotting and prothrombotic factors as well as physiologic anticoagulants. We recommend a prospective study to determine if the heightened associations seen in this study play a role in the pathogenesis of CLUs in SCA patients. If they do, it may be useful to find out if antiplatelet agents or/and anticoagulants will quicken the healing of CLUs in SCA patients [35].

## Figures and Tables

**Table 1:** General characteristics of study participants.

Group characteristics	SCA with CLU	SCA without CLU	HbAA	*P* value
Gender (M/F)	29/21	22/28	41/4	—
Age (years)	31.26 ± 7.87	25.14 ± 10.24	33.02 ± 7.91	0.001
Hb concentration (g/dL)	7.30 ± 1.42	7.99 ± 1.39	14.01 ± 1.29	0.001

Data are presented as mean ± standard deviation.

**Table 2:** Coagulation profile of study subjects.

Parameter	SCA with CLU	SCA without CLU	HbAA	*P* value (ANOVA)
Platelet count ( × 10^9^/L)	478.12 ± 177.32	424.20 ± 169.08	226.28 ± 53.18	0.001
PT(seconds)	16.02 ± 2.57	16.04 ± 1.68	14.91 ± 1.24	0.006
aPTT (seconds)	31.27 ± 6.16	34.51 ± 6.80	35.20 ± 6.06	0.006

Data are presented as mean ± standard deviation.

**Table 3:** Levels of fibrinogen and D-dimers among study subjects.

Characteristic	SCA with CLU	SCA without CLU	HbAA	*P* value (ANOVA)
Fibrinogen (mg/dl)	314.30 ± 109.83	284.90 ± 83.46	276.89 ± 88.02	0.127
D-dimer (*µ*g/ml)	1.56 ± 2.90	1.04 ± 1.56	0.52 ± 0.46	0.037

Data presented as mean ± standard deviation.

**Table 4:** Association between selected patients' characteristics and coagulation profile.

Parameter		Age	Duration of CLU	Frequency of CLU	Hb concentration	Platelet count
Age	R	1.000	0.351^*∗*^	0.108	0.182^*∗*^	−0.349^*∗*^
	P	—	0.015	0.455	0.028	0.000
Hb	R	0.182^*∗*^	−0.331^*∗*^	−0.204	1.000	−0.664^*∗*^
	P	0.028	0.021	0.156	—	0.000
Fibrinogen	R	0.096	0.131	−0.104	−0.008	−0.037
	P	0.253	0.374	0.471	0.922	0.659
D-dimer	R	0.065	−0.062	−0.024	−0.233^*∗*^	0.126
	P	0.440	0.676	0.867	0.005	0.130
PT	R	−0.076	0.035	−0.162	−0.233^*∗*^	0.241^*∗*^
	P	0.360	0.815	0.260	0.005	0.004
APTT	R	−0.096	−0.181	−0.086	0.185^*∗*^	−0.120
	P	0.253	0.219	0.552	0.026	0.149
Platelets	R	−0.349^*∗*^	−0.166	−0.311^*∗*^	−0.664^*∗*^	1.000
	P	0.000	0.261	0.028	0.000	—

*r*: coefficient of correlation; CLU: chronic leg ulcer; Hb: haemoglobin; PT: prothrombin time; aPTT: activated partial thromboplastin time, ^*∗*^Correlation is significant at the 0.05 level.

## Data Availability

The data supporting the findings in this study are available to researchers who meet the criteria for access to confidential data from the corresponding author upon request. This will be done after approval of the request by the ethics and protocol review committee, College of Health Sciences, University of Ghana.
